# PD-1 and PD-L1 expression on TILs in peritoneal metastases compared to ovarian tumor tissues and its associations with clinical outcome

**DOI:** 10.1038/s41598-021-85966-0

**Published:** 2021-03-18

**Authors:** Christine Bekos, Dietmar Pils, Sabine Dekan, Gerda Hofstetter, Peter Horak, Alexander Reinthaller, Stephan Polterauer, Richard Schwameis, Stefanie Aust

**Affiliations:** 1 Department of Obstetrics and Gynecology, Comprehensive Cancer Center (CCC), Medical University of Vienna, Vienna, Austria; 2 Division of General Surgery, Department of Surgery, Comprehensive Cancer Center (CCC), Medical University of Vienna, Vienna, Austria; 3 Department of Pathology, Medical University of Vienna, Vienna, Austria; 4 Division of Translational Medical Oncology, National Center for Tumor Diseases (NCT) Heidelberg and German Cancer Research Center (DKFZ), Heidelberg, Germany

**Keywords:** Ovarian cancer, Cancer immunotherapy

## Abstract

The therapeutic potential of immune checkpoint inhibitors is currently being investigated in epithelial ovarian cancer (EOC), but immunological effects of the programmed cell death protein 1 (PD-1)/programmed cell death 1 ligand 1 (PD-L1) axis in EOC still remain poorly understood. The aim of this study was thus to compare infiltration rates of PD-1 and PD-L1 expressing tumor infiltrating leucocytes (TILs) in primary ovarian tumor tissue and metastatic intraperitoneal implants and to investigate its impact on overall survival (OS). Tumor specimens (ovarian tumor tissues and intraperitoneal metastases) of 111 patients were used to investigate the PD-1, PD-L1 and CD8 expression rates on TILs and PD-L1 expression rate of tumor cells. The percentages of CD8, PD-1, and PD-L1 expressing subpopulations of TILs differ in primary ovarian tumor tissues and metastatic intraperitoneal implants. High PD-1 among TILs in peritoneal metastases were associated with favorable OS. High PD-L1 expression in TILs was associated with poor OS. Combining both factors in peritoneal metastases revealed an unfavorable prognosis. Primary ovarian tumor tissue and intraperitoneal metastatic tissues in EOC might have different strategies to evade immune control. Those findings are of importance for the process of biomarker assessment to predict patients’ response to immunotherapy.

## Introduction

Treatment for epithelial ovarian cancer (EOC) has recently been improved for a subset of patients due to extensive research on homologous recombination deficiency and associated therapeutic targets. Despite those achievements, EOC is mainly diagnosed at an advanced stage and recurrence rates as well as mortality are high and new therapeutic strategies are needed.

Immunotherapies can significantly prolong survival of a portion of patients with solid tumors, particularly hypermutated cancers such as melanoma, renal cancer, and non-small cell lung cancer^1–3^. A variety of immunotherapies have been investigated over the years, and still, little is known on how to best match the most promising approach to each individual patient following the paradigm of Precision Medicine^4^. Currently, the therapeutic potential of immune checkpoint inhibitors is being investigated in numerous clinical trials not only in the recurrent setting but already in first line treatment of EOC patients (e.g. NCT03737643, NCT03249142, NCT03602859 and NCT03522246). Though, compared to other solid tumors, the response rates reported so far seem to be modest, particularly as single-agent use^5,6^.

The functional impairment of T cells and the complex immunological effect of the programmed cell death protein 1 (PD-1) / programmed cell death 1 ligand 1 (PD-L1) axis in EOC remains poorly understood. One of the characteristics of EOC is an effortless tumor cell spread within the intraperitoneal cavity. The majority of tumors are diagnosed at an advanced stage with multiple intraperitoneal metastases, present as miliary or bulky lesions^7–9^ and just alike, recurrence predominantly occurs within the intraperitoneal cavity. It can be assumed, that the functional impairment of T-cell mediated immunity is special in ovarian cancer due to the complex microenvironment of the intraperitoneal ecosystem.

In a previous study we showed that the upregulation of PD-L1 on tumor cells is in an interplay with the down-regulation of tumor cell MHC I gene expression, thus highlighting several different immunological escape mechanisms in EOC^9^. The T-cell expression of PD-L1 on the other hand is less well-understood but assumed to induce intratumoral immune tolerance^10^. The upregulation of PD-1 on TILs induces a state of T cell exhaustion and impaired effector function^11^ but little is known on the impact of PD-1 and PD-L1 expressing TILs and the immune-tumor interaction in ovarian cancer and respective intraperitoneal metastatic tumor lesions.

The aim of this study was thus to map the infiltration rate of PD-1 and PD-L1 expressing TILs together with CD8 positive T cells among TILs in EOC, focusing on a comparison of primary ovarian tumor tissue with tissue from metastatic intraperitoneal tumor implants and the respective impact on patients 10 year survival.

## Results

### Study population

Tumor tissues of 111 EOC patients were analyzed in this study. These patients were diagnosed with and treated for EOC between 2004 and 2009 at the Medical University of Vienna (Comprehensive Cancer Center), Austria. Patients’ demographics are shown in Table 1.

**Table 1:** Patients’ characteristics.

Parameter	N (%) or median [Q1-Q3]^1^
Total number of patients enrolled	111
Age at diagnosis (years)	61.0 [49.0–70.5]
**Histological type**	
**Serous adenocarcinoma**	
LGSOC	24 (21.6%)
HGSOC	63 (56.8%)
Endometrioid adenocarcinoma	16 (14.4%)
Mucinous adenocarcinoma	6 (5.4%)
Clear cell carcinoma	2 (1.8%)
**Tumor stage**	
FIGO I	15 (13.5%)
FIGO II	6 (5.4%)
FIGO III	76 (68.4%)
FIGO IV	14 (12.6%)
**Residual tumor (one case missing and imputed as R1)**	
R1 (≥ 2 cm); R1 (Yes)	30 (27.0%); 47 (42.3%)
R0 (< 2 cm); R0 (No)	81 (73.0%); 64 (57.7%)
**Source of tissue**	
Primary tumors only	44 (39.6%)
Implants only	14 (12.6%)
Paired primary and implant	53 (47.8%)

^1^Q1, 25th and Q3, 75th percentiles.

Median age of the EOC patients at time of cytoreductive surgery was 61.0 years. The median observation period was 45.1 months (min 0.2, Q1 23.9, Q3 108.0, max 178.4 months). Within the observation period, 69 patients died (62.2%).

### Immunohistochemistry

Staining for CD8, PD-1 and PD-L1 was performed on formalin fixed and paraffin-embedded tissue microarrays (TMAs) with tissues from the primary ovarian tumor and peritoneal metastases from the intraperitoneal cavity^12^.

**Figure 1:**
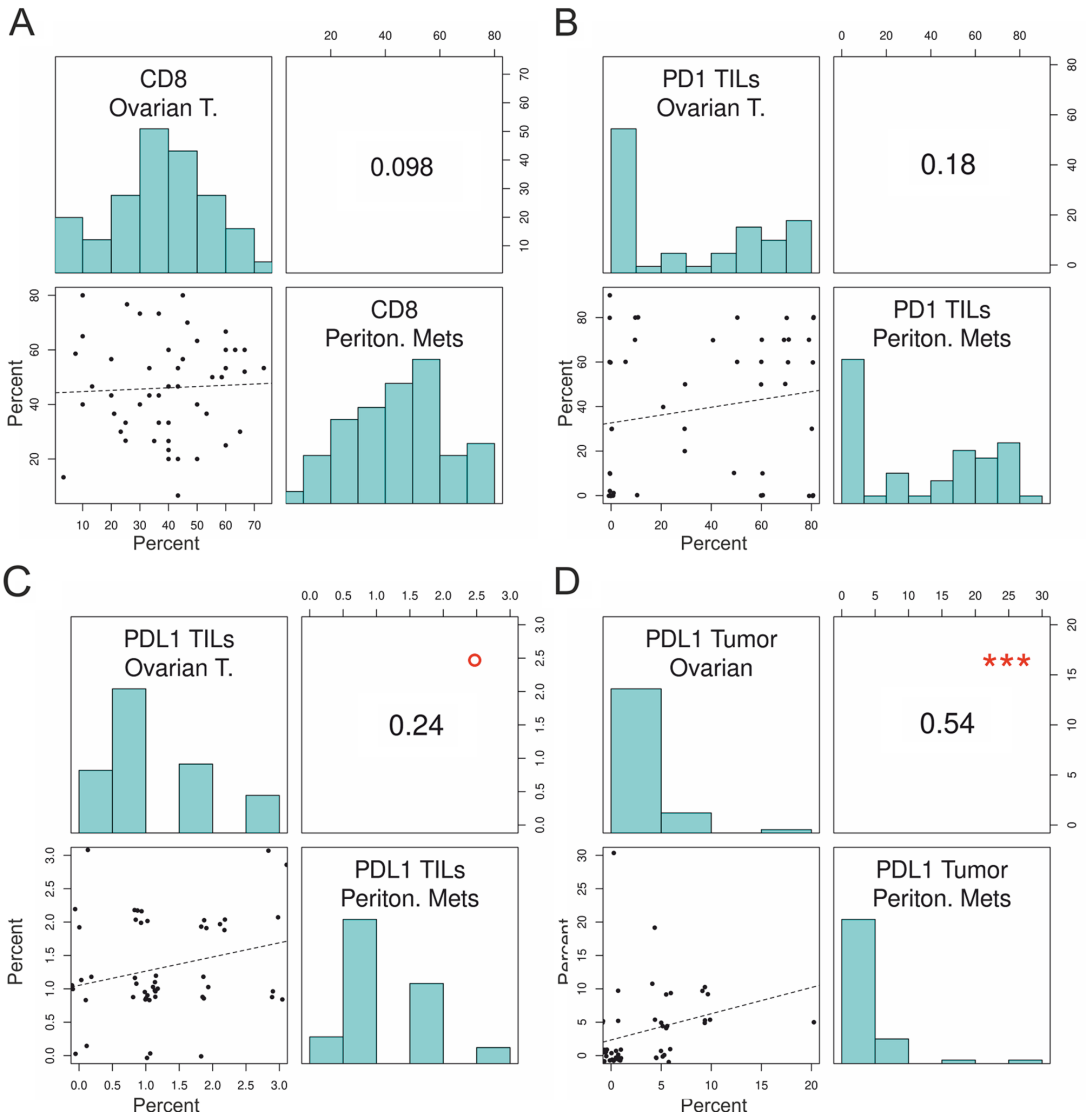
Correlation of CD8 TILs (**A**), PD-1 TILs (**B**), PD-L1 TILs (**C**) and PD-L1 Tumor (**D**) in ovarian tumor tissue with the respective values in peritoneal metastases. °p < 0.1; ***p < 0.001. The top left and bottom right quadrants show histograms of the distribution of percentages. X-axis for the left quadrants is shown at the bottom and for the right side at the top of each panel. For Plots (**B**), (**C**), and (**D**) values were shown around the categorized percentages to see all patients.

CD8 positive TILs were assessed on ovarian tissues and peritoneal metastases by immunohistochemistry. Results were given in % CD8 + TILs and the mean of three cores for each case was calculated. The CD8 expression in TILs on ovarian tissue did not correlate with that in peritoneal metastases (Table 2, Fig. 1).

**Table 2:** Correlation between percentages of positive cells of PD-1 among TILs, PD-L1 among tumor cells and TILs, and CD8 positive cells among TILs in paired primary tissues and implants.

	Ovarian tissues	Peritoneal metastases	Spearman’s rank corr. coefficient	P-Value^1^
**CD8 TILs**	38.18 (19.69)	45.25 (17.87)	**0.098**	**0.494**
PD-1 TILs	36.31 (33.62)	34.82 (32.60)	**0.182**	**0.205**
PD-L1 TILs	1.15 (0.88)	1.27 (0.73)	**0.242**	**0.090**
PD-L1 Tumor	2.66 (3.79)	3.48 (5.33)	**0.540**	** < 0.001**

Given are mean (SD), ^1^ According Spearman’s rank correlation.

Data on PD-1 expression in TILs were available for 103 patients and given in % PD-1 positive TILs. The strongest PD-1 expression of the three cores of each case was used for further statistical analyses. The PD-1 expression in TILs on ovarian tissue did not correlate with that in peritoneal metastases.

PD-L1 expression in TILs and tumor cells were assessed in all 111 patients. The strongest PD-L1 expression of the three cores of each case was used for further statistical analyses. Results in ovarian tissue did not correlate with peritoneal metastases. PD-L1 expression in tumor cells in ovarian tissue correlated directly with that in peritoneal metastases (p < 0.001).

**Figure2:**
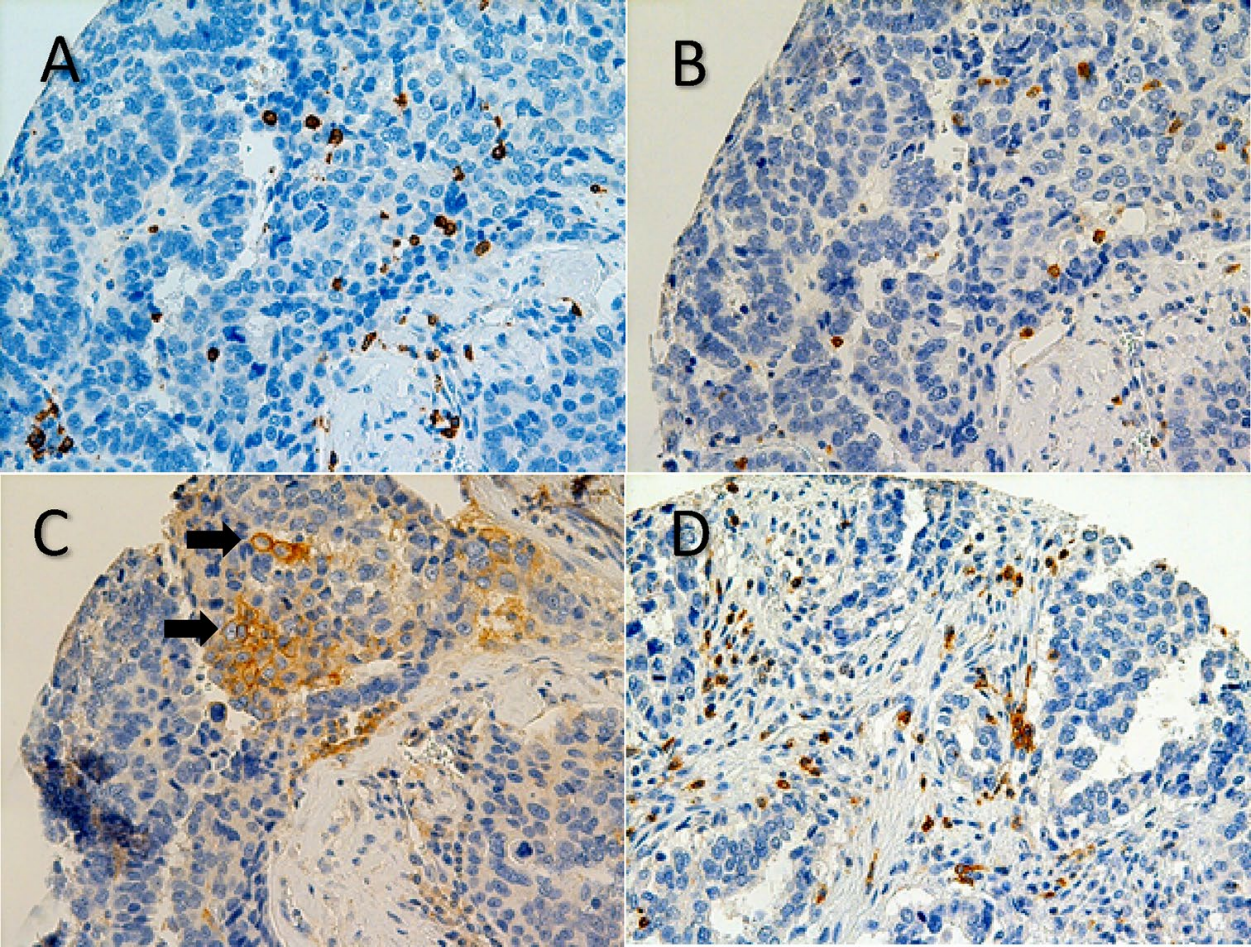
(**A**) Representative immunohistochemistry (IHC) of CD8 in high grade serous ovarian cancer, 40$\times$: CD8 positive tumor infiltrating lymphocytes (TILs) (**B**) IHC of PD-L1, 40$\times$: PD-L1 positive TILs. (**C**) IHC of PD-L1, 40$\times$: tumor cells stain positively for PD-L1, membrane staining was observed. (**D**) IHC of PD-1, 40$\times$: PD-1 positive TILs.

Representative IHC staining for CD8 positive TILs, PD-L1 positive TILs, PD-L1 positive tumor cells and PD-1 positive TILs are shown in Fig. 2. In tumor cell staining for PD-L1, a membranous staining was observed.

### Survival analyses

**Figure 3:**
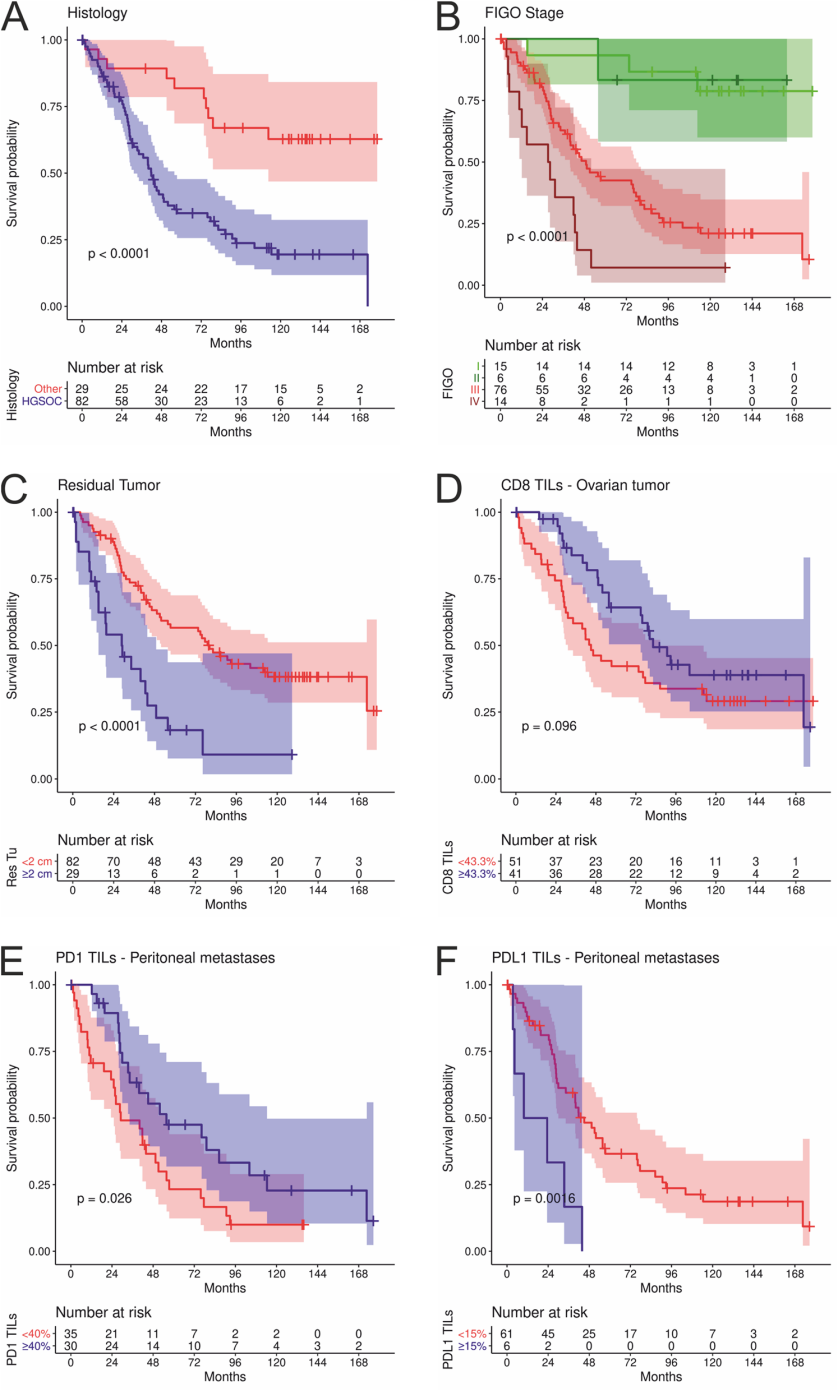
Kaplan–Meier estimates for the clinicopathologic parameters histology (**A**), FIGO stage (**B**), and residual tumor mass after debulking surgery (**C**) and optimally dichotomized CD8 positive TILs in ovarian tumors (**D**), optimally dichotomized PD1 positive TILs (**E**) and optimally dichotomized PDL1 positive TILs in peritoneal metastases (**F**). P-values according to Log-Rank tests.

In univariate Cox regression survival analysis, higher age (p < 0.001), HGSOC (p < 0.001), advanced FIGO stage (p < 0.001) and residual tumor mass after debulking surgery (p < 0.001) were associated with poor overall survival. Kaplan–Meier estimates are shown in Fig. 3A–C. Despite the fact that in clinical practise total resection (no residual tumor) is the accepted standard, in this cohort a cut-off of 2 cm versus < 2 cm showed a more significant impact and survived all backward selection procedures in all multiple Cox regression analyses shown below, therefore this cut-off was used. In a multiple Cox regression survival analysis, higher age (p < 0.001), advanced FIGO stage (p < 0.001) and residual tumor ≥ 2 cm (p = 0.003) were associated with poor overall survival (Table 3).

**Table 3:** Univariate and multiple Cox regression overall survival analyses in 111 patients with ovarian cancer.

N = 111 69 events	Overall survival			
	Univariate		Multiple	
	HR [CI_95_]	*P*-value	HR [CI_95_]	*P*-value
Age (decades)	1.59 [1.31–1.94]	< 0.001	1.84 [1.44–2.34]	< 0.001
Histology (HGSOC > non HGSOC)	3.74 [1.90–7.38]	< 0.001	2.86 [1.35–6.07]	0.006
FIGO (IV > III > II > I)	2.71 [1.82–3.91]	< 0.001	1.97 [1.31–2.94]	0.001
Residual tumor (R1 > R0)*	3.23 [1.85–5.34]	< 0.001	2.20 [1.27–3.76]	0.004

CI_95_, 95% confidence interval. *Using a cut-off of 0 cm for R1 in the multiple Cox regression model showed no significant independent impact on OS any more (HR 1.44, p = 0.170).

In multiple Cox regression models raw percentage values of CD8 + TILs, PD-1 + TILs, PD-L1 + TILs and PD-L1 + tumor cells were analyzed individually always together with known clinicopathologic factors (age, histology, FIGO stage, and residual tumor mass after debulking surgery) in ovarian tumor tissue and peritoneal metastases, respectively (Supplement; S-Table 1).

After backward selection minimizing the Akaike information criterion, CD8 percentages in ovarian tissues and PD-1 and PD-L1 percentages of TILs in peritoneal metastases remained in the respective multiple Cox regression models. PD-L1 expression on tumor cells revealed no significant impact on overall survival. Due to the explorative approach of this study we defined cut-off values to further evaluate the prognostic significance of the analyzed parameters. To define an optimal prognostic cut-off an algorithm was used that simultaneously included clinical parameters and was described in detail in the methods section. After defining optimal cut-offs for CD8 (i.e. 44.3%) among TILs in ovarian tissues, PD-1 (i.e. 40%) and PD-L1 (i.e. 15%) among TILs in peritoneal metastases as well as dichotomization according to these cut-offs, univariate and multiple Cox regression models were performed (Fig. 3D–F and Tables 4, 5, 6).

**Table 4:** Univariate and multiple Cox regression overall survival analyses for CD8 of TILs percentages in ovarian tissues.

	Overall survival			
	Univariate		Multiple	
	HR [CI_95%_]	*P*-value	HR [CI_95%_]	*P*-value
CD8 TILs (≥ 43.3% vs. < 43.3%)	0.64 [0.37–1.09]	0.098	0.52 [0.29–0.91]	0.023
Age (decades)	1.64 [1.33–2.02]	< 0.001	1.86 [1.41–2.46]	< 0.001
Histology (HGSOC > Other)	4.11 [2.00–8.45]	< 0.001	3.97 [1.72–9.19]	0.001
Figo (IV > III > II > I)	2.55 [1.70–3.83]	< 0.001	1.84 [1.23–2.76]	0.003
Residual tumor mass (R1 > R0)	2.91 [159–5.31]	< 0.001	2.24 [1.21–4.17]	0.011

Combined hazard ratios and p-values for the dichotomized CD8 expression of TILs in univariate Cox-regressions (left) or multiple Cox-regressions, corrected for age, FIGO stage, and residual tumor mass (right). HR, Hazard Ratio; 95%CI, 95% Confidence Interval; OS, overall survival.

After dichotomization we found 51 patients with < 43.3% and 41 with ≥ 43.3% CD8 + TILs in ovarian tissues, respectively. We found 35 patients with < 40% PD-1 positive TILs and 30 with ≥ 40% in peritoneal metastases, respectively. We found 61 patients with < 15% PD-L1 positive TILs and 6 with ≥ 15% in peritoneal metastases, respectively.

In EOC patients, higher PD-1 TIL and lower PD-L1 TIL percentages in peritoneal metastases showed a significant impact on OS (p = 0.003 and p = 0.043, respectively) (Fig. 3E,F, Tables 5 and 6)). This was always independent of histology.

**Table 5:** Univariate and multiple Cox regression overall survival analyses of PD-1 of TILs percentages in peritoneal metastases.

	Overall survival			
	Univariate		Multiple	
	HR [CI_95%_]	*P*-value	HR [CI_95%_]	*P*-value
PD-1 TILs (≥ 40% vs. < 40%)	0.52 [0.29–0.93]	0.028	0.40 [0.22–0.75]	0.004
Age (decades)	1.50 [1.15–1.97]	0.003	1.64 [1.22–2.19]	< 0.001
Histology (HGSOC > Other)	2.62 [1.03–6.67]	0.043	n.i	
Figo (IV > III > II > I)	3.77 [2.03–7.00]	< 0.001	4.21 [2.18–8.14]	< 0.001
Residual tumor mass (R1 > R0)	1.69 [0.91–3.15]	0.096	2.35 [1.18–4.67]	0.015

Combined hazard ratios and p-values for the dichotomized PD-1 expression in TILs in univariate Cox-regressions (left) or multiple Cox-regressions, corrected for age, FIGO stage, and residual tumor mass (right). HR, Hazard Ratio; CI_95%_, 95% Confidence Interval; OS, overall survival; n.i., not included; according selection by the backward selection method minimizing the Akaike Information Criterion (AIC).

**Table 6:** Univariate and multiple Cox regression overall survival analyses of PD-L1 of TILs percentages in peritoneal metastases.

	Overall survival			
	Univariate		Multiple	
	HR [CI_95%_]	*P*-value	HR [CI_95%_]	*P*-value
PD-L1 TILs (≥ 15% vs. < 15%)	3.79 [1.56–9.23]	0.003	3.00 [1.05–8.72]	0.041
Age (decades)	1.46 [1.12–1.91]	0.005	1.67 [1.24–2.24]	< 0.001
Histology (HGSOC > Other)	2.79 [1.00–7.83]	0.051	n.i	
Figo (IV > III > II > I)	3.61 [1.95–6.65]	< 0.001	3.07 [1.56–6.03]	0.001
Residual tumor mass (R1 > R0)	1.66 [0.90–3.08]	0.106	1.61 [0.83–3.11]	0.160

Combined hazard ratios and p-values for the dichotomized PD-L1 expression in TILs in univariate Cox-regressions (left) or multiple Cox-regressions, corrected for age, FIGO stage, and residual tumor mass (right). HR, Hazard Ratio; CI_95%_, 95% Confidence Interval; OS, overall survival; n.i. = not included; according selection by the backward selection method minimizing the Akaike Information Criterion (AIC).

Combining both factors in peritoneal metastases revealed an unfavorable prognosis (*cf.* Fig. 2D): PD-1high/PD-L1low $\to$ PD-1low/PD-L1low $\to$ PD-1high/PD-L1high $\to$ PD-1low/PD-L1high, with the worst survival.

If using an R1 cut-off of 0 cm for all of these analyses, residual tumor was removed from all models by the backward selection procedure (was thus not independent relevant for OS) except for the model with CD8 percentages in the primary tumors. The following HRs and p-values were obtained (without correction for residual tumor with 2 cm cut-off): CD8 TILs primary tumor: HR 0.57, p = 0.0519; PD-1 TILs metastases: HR 0.47, p = 0.0133; PD-L1 TILs metastases: HR 3.47, p = 0.0160; and combined as consecutive factor as above: HR 0.59, p = 0.0023.

In a next step survival analyses were performed in the subgroup of advanced stage HGSOC. In this subgroup, a significant impact of PD-1 TIL expression on OS was seen univariately (Fig. 2 in supplemental material) and corrected for age, FIGO stage, and residual tumor mass (p = 0.037). A lower percentage of tumor infiltrating PD-L1 positive lymphocytes was associated significantly with favorable OS univariately without reaching significance if corrected for age, FIGO stage, and residual tumor mass (p = 0.084) (Fig. 2 in supplemental material).

## Discussion

In 111 EOC patients we found no significant correlations between the respective percentages of CD8, PD-1, and PD-L1 expressing TILs in ovarian tumor tissues compared to intraperitoneal metastatic tissues obtained at primary debulking surgery. Only PD-L1 expression on tumor cells correlated significantly between ovarian tissues and peritoneal metastases. We thus showed for the first time that the percentages of CD8, PD-1, and PD-L1 expressing subpopulations of TILs differ in primary ovarian tumor tissues and metastatic intraperitoneal tumor implants. Our findings complement recently published data on a significantly higher number of tumor infiltrating CD8 positive as well as a lower number of PD-1 positive immune cells in metastatic compared to primary lesions^13^. Additionally, in line with several previously published studies^14–17^ our results confirm the favorable prognosis in patients with higher levels of tumor infiltrating CD8 + T cells in ovarian cancer tissue. As novel finding we could show that higher PD-1 TIL and lower PD-L1 TIL percentages in peritoneal metastases had a significant impact on patients’ survival. Further, a recent study observed that PD-L1 immunostaining in tumor cells and stromal tumor-infiltrating lymphocytes was associated with an increased overall survival^18^.

In EOC, intraperitoneal tumor spread is not only a major characteristic of the tumors metastatic behavior but often limits surgical success^19^ and thus directly impairs patients’ survival and quality of life^20^. We are not the first to address tumor heterogeneity between primary ovarian tissue and metastatic lesions. Heterogeneity has been pointed out particularly on the genomic and proteomic levels^21–24^ and needs to be considered also on the immunological level^25^, particularly in the light of immunotherapy development^13^.

Considering the different percentages of each analyzed marker determined in ovarian tumor tissues and peritoneal metastases, overall survival analyses were performed for both tissue origins individually. High CD8 among TILs frequencies in ovarian tissues and high PD-1 among TILs frequencies in peritoneal metastases were associated with favorable survival. Even though the upregulation of PD-1 on T cells has emerged as a major marker of T cell dysfunction^26^, high PD-1 expression has been shown to be associated with favorable prognosis in ovarian cancer in a variety of studies, including a pooled analysis with large-scale public cohorts of samples comprising 13 studies on HGSOC^27,28^. Webb JR et al. investigated the functional status of PD-1 positive TILs in EOC and hypothesized, that in ovarian cancer, in contrast to many other tumor entities, PD-1 expression is rather indicating T cell activation than exhaustion^29^. Of note, PD-1 expression was not seen on ovarian cancer tumor cells, contrary to a previous publication showing unexpectedly high PD-1 expression in tumor cells^28^ but consistent with other publications using approved PD-1 antibodies^29,30^.

High PD-L1 among TILs frequencies was associated with unfavorable survival, with a relative higher HR compared to the two factors above. Combining both factors in peritoneal metastases revealed that patients with PD-1^high^/ PD-L1^low^ infiltration rates had the longest median OS with a declining prognosis in patients classified as PD-1^low^/PD-L1^high^, with the worst survival. Most studies on PD-L1 expression and its impact on survival in EOC focused particularly on PD-L1 expression on tumor cells or macrophages^31^. Differences in PD-L1 tumor cell expression were observed comparing primary tumor tissue to tumor tissues acquired in the setting of tumor recurrence (or consecutive recurrences) revealing a significant increase of PD-L1 in relapsed serous EOC^9^. PD-L1 signaling in T cells on the other hand still needs to be further investigated. Recently, it has been discovered, that T cell expression of PD-L1 results in a suppressive intracellular signaling induction and creation of intra-tumoral immune tolerance^10^. Additionally, the authors showed that PD-L1 expressing T cells suppress neighboring PD-1 positive T cells^10^. This interplay might be related to our finding of an increased prognostic significance of a combination of a high PD-1 and low PD-L1 percentage of TILs in metastatic EOC tumor tissue.

The need to define new therapeutic approaches for EOC patients and the advances in immunotherapy with remarkable success in selected cancer entities that could not be reached in EOC so far, highlight the need for a critical rethinking of possible new immunotherapy strategies. An understanding of the processes that drive and maintain different dysfunctional T cell states is essential. Our findings support further studies of immune regulatory mechanisms in peritoneal metastases in EOC.

Limitations of the presented study include a limited patient number and a retrospective collection of primary and metastatic tumor tissue. Additionally, colocalization staining of multiple immunohistochemical markers and expression of various immune cells could not be performed but will be developed within future projects.

Still, our data strenghten the assumption that within EOC, primary and metastatic tumor tissues might have evolved different independent strategies to evade immune control. Independent immunological strategies observed in primary or metastatic as well as primary or recurrent tumor tissues need to be considered also in the process of biomarker assessment to predict patients’ response to immunotherapy. A novel clinical immunotherapy-trial design based on peritoneal sampling and the search for specific biomarkers representing peritoneal immune response could be reconsidered to better understand the role of immunomodulators in ovarian cancer. Furthermore, sampling of EOC at several distinct locations might improve the prognostic accuracy in biomarker development.

## Material and methods

### Study population

Formalin-fixed and paraffin-embedded (FFPE) surgical specimens from 111 consecutive patients diagnosed with and treated for EOC between 2004 and 2009 at the Comprehensive Cancer Center Vienna were used in the present study (Table 1). Depending on availability, paired samples of primary ovarian tumor tissue and intraperitoneal metastatic tissues, only ovarian or only metastatic tumor tissues had been collected retrospectively. Demographic data were retrospectively extracted from medical records. The study was performed as recommended by the guidelines of the Declaration of Helsinki. The study was approved by the Ethics Committee of the Medical University of Vienna (IRB approval number: 1678/2014) before the study was initiated. An informed consent was obtained from all patients. All patients were treated in accordance with the standards of our institution with upfront surgery and adjuvant platinum-based chemotherapy. Surgical staging according to FIGO guidelines was performed, including hysterectomy, bilateral salpingo-oophorectomy, pelvic and/or para-aortic lymphadenectomy, appendectomy, omentectomy, and cytoreductive procedure to resect all gross tumor masses, as previously described^12^. Residual tumor load was defined as < 2 cm or ≥ 2 cm. Overall survival was the time interval between diagnosis and cancer-associated death. Patients without death were censored at the time of last follow-up.

Low grade serous ovarian cancer was defined as serous ovarian cancer with grading 1. High grade serous ovarian cancer was defined as serous ovarian cancer with grading 2 and 3.

### Immunohistochemistry

Staining was performed on paraffin-embedded tissue microarrays (TMAs). The TMAs were assembled by taking 3 core needle biopsies (core dimension 1 mm) from defined tumor regions in the paraffin embedded tumor tissue blocks using techniques and an apparatus developed by Beecher Instruments Inc, Micro-Array Technology (Sun Prairie, WI, USA)^12^. Each tumor tissue sample was treated under the same conditions and the whole cohort could be analyzed on two slides. Tissues from ovarian tumor tissue (P, for “primary”) and from metastatic implants within the peritoneal cavity (M, for “metastasis”) were employed for immunohistochemistry (IHC)^9^. IHC procedures were performed at room temperature. Four-micrometer sections were mounted onto highly adhesive slides (Thermo Scientific SuperFrost 73 Ultra Plus; Thermo Fisher Scientific).

In brief, staining was performed using a validated anti-PD-L1 antibody (PD-L1 clone E1L3N XP Rabbit mAb, Cell Signaling Technology, Danvers, MA, USA). This antibody has shown more reliable results compared to previously described anti-PD-L1 antibodies. In a previous study of our team we tested the accuracy of this specific PD-L1 antibody comparing it to another one (VENTANA PD-L1 (SP263) Rabbit Monoclonal Primary Antibody) and could demonstrate a highly similar staining pattern^9^. Additionally, staining for PD-1 and CD8 was performed using the monoclonal mouse anti-PD-1 antibody (MRQ-22) from Cell Marque and the anti-CD8 monoclonal mouse antibody (Clone C8/144B) from Dako^14^. IHC was performed in a standardized setting using the Leica bond Polymer Refine Detection kit DS9800 according to staining procedures established at the Department of Pathology, Medical University of Vienna. Omission of primary antibody served as negative control.

Percentages of PD-L1 positive EOC tumor cells and TILs, percentages of CD8 positive cells, and PD-1 positive TILs among total CD8 + cells were analyzed.

Cases were scored individually by two observers, including a pathologist specialized in gyneco-oncology, blinded to clinical parameters. Lymphocytes were determined by colocalized CD8 staining.

### Data analysis and statistics

Data analysis was performed using GNU R 3.6.0 and R-packages described below. The one missing information for residual tumor mass after debulking surgery was imputed using R-package mice 3.8.0^32^ and all clinicopathologic information as predictors. In order to compare percentages of CD8 TILs, PD-1 TILs, PD-L1 TILs, and PD-L1 tumor cells between ovarian tissue and peritoneal metastases Spearman’s rank correlation analyses were performed. P-values of < 0.05 were considered statistically significant. With respect to overall survival, continuous and dichotomized percentage values were analyzed using univariate and multiple Cox regression analyses.

After analyzing raw percentage values with known clinicopathologic factors in multiple Cox regression models, backward selection minimizing the Akaike information criterion was performed using R-package MASS 7.3–51.6 (function *stepAIC*, direction = “backward”)^33^.

In a next step, the final Cox regression models were used to define the optimal cut-off percentages for the three factors with R-package SurvMisc 0.5.5 (function *cutp*)^34^ using the multiple Cox regression models, thus correcting for clinicopathologic factors indicated in Table 1 in supplemental material in bold: CD8 among TILs in ovarian tissues, 44.3%; PD-1, 40%; and PD-L1, 15% among TILs in peritoneal metastases. After dichotomization according given values (always ≥ versus <) final univariate and multiple Cox regression models were performed using all clinicopathologic factors initially remained in the Cox regression models by the backward selection procedure described above (Tables 4, 5 and 6).

## Supplementary information

Supplementary information.

Supplementary figure 1.

Supplementary figure 2.
