# Supplementary material for: PD-1 and PD-L1 expression on TILs in peritoneal metastases compared to ovarian tumor tissues and its associations with clinical outcome
Source: Sci Rep. 2021 Mar 18;11:6400. doi: 10.1038/s41598-021-85966-0 (PMC7973418; doi:10.1038/s41598-021-85966-0)
Supplement: Supplementary file 1 — Supplementary information. [file 41598_2021_85966_MOESM1_ESM.docx]

**PD-1 and PD-L1 expression on TILs in peritoneal metastases compared to ovarian tumor tissues and its associations with clinical outcome**

**Bekos Christine^1^, Pils Dietmar^2^, Dekan Sabine^3^, Hofstetter Gerda^3^, Horak Peter^4^, Reinthaller Alexander^1^, Polterauer Stephan^1^, Schwameis Richard^1^, Aust Stefanie^1^***

^1^ Department of Obstetrics and Gynecology, Comprehensive Cancer Center (CCC), Medical University of Vienna, Austria

^2^ Division of General Surgery, Department of Surgery, Comprehensive Cancer Center (CCC), Medical University of Vienna, Austria

^3^ Department of Pathology, Medical University of Vienna, Austria

^4^ Division of Translational Medical Oncology, National Center for Tumor Diseases (NCT) Heidelberg and German Cancer Research Center (DKFZ), Heidelberg, Germany

* Corresponding Author:

Stefanie Aust

E-mail: stefanie.aust@meduniwien.ac.at

**Supplemental material**

**Table 1.** Univariate and multiple Cox regression analyses of analytes with clinicopathologic parameters in ovarian tissue and peritoneal metastases.

| Ovarian Tissues | | | | | | | | |
| --- | --- | --- | --- | --- | --- | --- | --- | --- |
| Analyte | | | | | Correcting factor | | | |
|  |  |  |  |  | Age | Histology | FIGO | ResT |
| N=97 | Univariate | | Multiple Cox regression | | | | | |
| 57 events | HR | P-value | HR | P-value | HR | HR | HR | HR |
| **CD8 TILs** | 1.00 | 0.790 | **0.98** | **0.050** | **1.89*** | **3.80*** | **1.91*** | **2.23*** |
| PD-1 TILs | 1.00 | 0.549 | 1.00 | 0.710 | 2.08* | 2.12 | 2.48* | 2.28* |
| PD-L1 TILs | 1.00 | 0.844 | 1.03 | 0.283 | 1.99* | 3.43* | 2.05* | 2.44* |
| PD-L1 Tumor | 1.03 | 0.449 | 1.00 | 0.851 | 1.86* | 3.49* | 1.98* | 2.25* |
|  |  |  |  |  |  |  |  |  |
| Peritoneal Metastases | | | | | | | | |
| Analyte | | | | | Correcting factor | | | |
|  |  |  |  |  | Age | Histology | FIGO | ResT |
| N=69 | Univariate | | Multiple Cox regression | | | | | |
| 51 events | HR | P-value | HR | P-value | HR | HR | HR | HR |
| CD8 TILs | 1.00 | 0.756 | 1.00 | 0.656 | 1.67* | 2.32 | 3.49* | 1.83 |
| **PD-1 TILs** | 0.99 | 0.051 | **0.99** | **0.041** | **1.71*** | 2.08* | **3.42*** | **2.08*** |
| **PD-L1 TILs** | 1.04 | 0.169 | **1.06** | **0.062** | **1.83*** | 2.38 | **2.92*** | **1.69** |
| PD-L1 Tumor | 1.09 | 0.005 | 1.04 | 0.293 | 1.68* | 2.17 | 3.05* | 1.73 |
|  |  |  |  |  |  |  |  |  |

*P-value <0.05. HR, Hazard Ratio; ResT, Residual Tumor;

**Figure 1.** Survival curves of the multiple Cox regression model for (A) CD8 of TILs percentages in ovarian tissues (≥43.3% versus <43.3%), (B) PD-1 of TILs percentages in peritoneal metastases (≥40% versus <40%), (C) PD-L1 of TILs percentages in peritoneal metastases (≥15% versus <15%), (D) combining both factors PD-1 and PD-L1 in peritoneal metastases. As these survival curves represent multiple Cox models, no censored patients are indicated.

**Figure 2.** Kaplan-Meier estimates for optimally dichotomized PD-1 positive TILs (A) and optimally dichotomized PD-L1 positive TILs (B) in advanced HGSOC peritoneal metastases. P-values according Log-Rank tests. Survival curves of the multiple Cox regression model for PD-1 positive TILs in advanced HGSOC peritoneal metastases and (B) PD-L1 positive TILs in advanced HGSOC peritoneal metastases . As these survival curves represent multiple Cox models, no censored patients are indicated. P-values according the multiple Cox regression models.
