# Supplementary figures and images for: PD-1 and PD-L1 expression on TILs in peritoneal metastases compared to ovarian tumor tissues and its associations with clinical outcome
Source: Sci Rep. 2021 Mar 18;11:6400. doi: 10.1038/s41598-021-85966-0 (PMC7973418; doi:10.1038/s41598-021-85966-0)

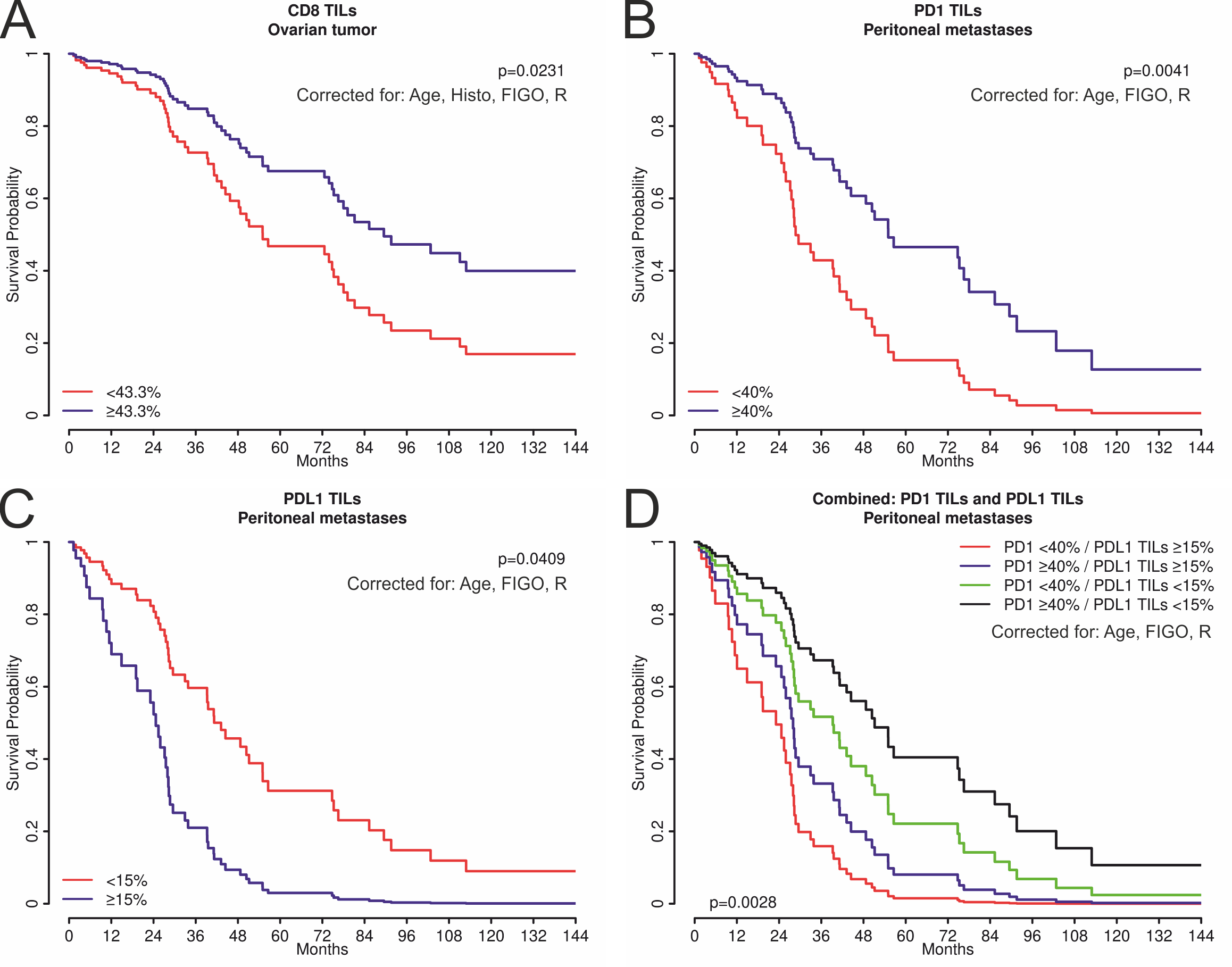

Supplement: Supplementary file 2 — Supplementary figure 1. [file 41598_2021_85966_MOESM2_ESM.png]

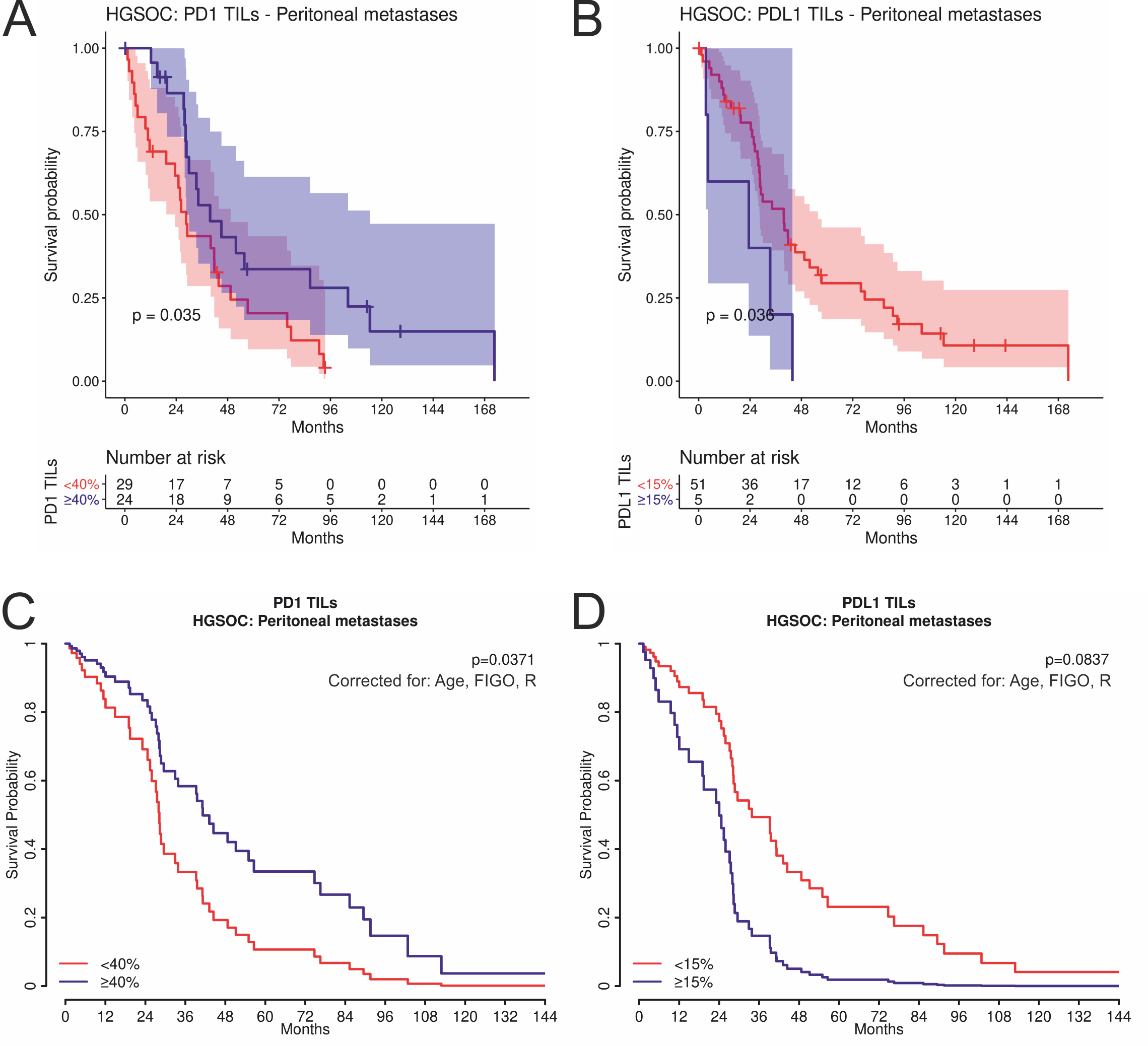

Supplement: Supplementary file 3 — Supplementary figure 2. [file 41598_2021_85966_MOESM3_ESM.png]
